# Letter from Persia

**Published:** 1847-02

**Authors:** A. H. Wright

**Affiliations:** Oroomiah


*Letter from Persia.—Leeches in Intermittent Fever, &c.*

We have just heard that the cholera has broken out in Teheran, the capital, though we have no definite information yet of the extent of its ravages. A physician residing in Tabreez, writes me, under date of August 1st, in the following manner: "We are all consternation here, at present, at the news of the cholera being at Teheran. The Prince (Governor of this part of Persia,) is most anxious to take all sanitary measures to prevent its arrival here, and has had communication with Dr. C. and myself on the subject, since which all the Rabab manufactories (cook-shops) have been sent outside of the town, and the selling of fruit diminished. Cleanliness is strictly enjoined, and a fresh supply of water let into the town from the gardens for watering the streets." The disease marched through these parts some twelve or fifteen years ago, and made dreadful havoc of human life. In many cases the natives, when attacked by it, used to throw themselves into a fountain or stream of cold water. The testimony is, as you would expect in such cases, that some lived and some died. By the way, what do you think of Andral's summary of the cholera? "Anatomical characters, insufficient; causes, mysterious; nature, hypothetical; symptoms, characteristic; diagnosis, easy; treatment, doubtful."

Are you familiar with the use of leeches, applied over the region of the spleen, in obstinate cases of intermittent fever? I have tried this treatment in two very bad cases, and with the most complete success. A Roman Catholic priest, a native of France, had been my patient some time, and I had become almost discouraged in using quinine and arsenic. He became worse and worse, and, dropsical symptoms supervening, his friends thought he would certainly die, and I began to think so too. In this state, I heard that Dr. Bell, Physician to the British Embassy at the Court of Persia, had used leeches in similar cases with great success, and I determined to try them on my patient. Dr. Bell's mode is to apply them on the day the moon fulls, and to repeat them every full moon until the disease is conquered. Though I had, of course, little confidence that the moon had any thing to do in the matter, I made the experiment exact to the letter, and at the first full moon applied twelve leeches over the spleen, intending to repeat the application at the next full moon in case the disease did not give way. But to my delight, and, I may add, surprise too, the poor priest, sallow and dropsical as he was, began to recover from that day, and I had no occasion to repeat the leeches.

I recently made a professional visit to Badr Rhem Bey, the celebrated Roordish chief of Buhtem on the river Tigris. His son, a youth of 12 or 14 years of age, had been suffering for about a year and a half from ague and fever of the *quartan* kind. He was reduced to a state which gave much uneasiness to his friends. At first I tried a purge and quinine, but without success. Indeed his next attack was more severe than before he had taken the medicine. I concluded to resort to the use of leeches, though some of his friends thought he would certainly die, if he were to lose blood, pale and emaciated as he was. Without regarding the moon as in the former case, I applied six leeches the day before he expected a paroxysm, and on the morning of the next day, gave him two doses of quinine of four grains each. The disease was broken from that time. What comments have you to make on these cases?

Many, many thanks for your Journal. It is always *very* acceptable.

I remain, my dear sir, yours very truly,

A. H. Wright.

*Oroomiah,* August 12, 1846. *Ibid.*

